# Novel Cyclic Peptides for Targeting EGFR and EGRvIII Mutation for Drug Delivery

**DOI:** 10.3390/pharmaceutics14071505

**Published:** 2022-07-20

**Authors:** Olga Furman, Alisa Zaporozhets, Dror Tobi, Andrii Bazylevich, Michael A. Firer, Leonid Patsenker, Gary Gellerman, Bat Chen R. Lubin

**Affiliations:** 1 Department of Chemical Engineering, Biotechnology and Materials, Ariel University, Ariel 40700, Israel; olgaf@ariel.ac.il (O.F.); firer@ariel.ac.il (M.A.F.); 2 Agriculture and Oenology Department, Eastern Regional R&D Center, Ariel 40700, Israel; 3 Department of Chemical Sciences, Ariel University, Ariel 40700, Israel; aliska.zaporozhets@gmail.com (A.Z.); andriib@ariel.ac.il (A.B.); leonidpa@ariel.ac.il (L.P.); garyg@ariel.ac.il (G.G.); 4 Adelson School of Medicine, Ariel University, Ariel 40700, Israel; drorto@ariel.ac.il; 5 Department of Molecular Biology, Ariel University, Ariel 40700, Israel; 6 Ariel Center for Applied Cancer Research, Ariel 40700, Israel

**Keywords:** EGFR, NSCLC, peptide, targeted drug delivery, glioblastoma

## Abstract

The epidermal growth factor–epidermal growth factor receptor (EGF-EGFR) pathway has become the main focus of selective chemotherapeutic intervention. As a result, two classes of EGFR inhibitors have been clinically approved, namely monoclonal antibodies and small molecule kinase inhibitors. Despite an initial good response rate to these drugs, most patients develop drug resistance. Therefore, new treatment approaches are needed. In this work, we aimed to find a new EGFR-specific, short cyclic peptide, which could be used for targeted drug delivery. Phage display peptide technology and biopanning were applied to three EGFR expressing cells, including cells expressing the EGFRvIII mutation. DNA from the internalized phage was extracted and the peptide inserts were sequenced using next-generation sequencing (NGS). Eleven peptides were selected for further investigation using binding, internalization, and competition assays, and the results were confirmed by confocal microscopy and peptide docking. Among these eleven peptides, seven showed specific and selective binding and internalization into EGFR positive (EGFR+ve) cells, with two of them—P6 and P9—also demonstrating high specificity for non-small cell lung cancer (NSCLC) and glioblastoma cells, respectively. These peptides were chemically conjugated to camptothecin (CPT). The conjugates were more cytotoxic to EGFR+ve cells than free CPT. Our results describe a novel cyclic peptide, which can be used for targeted drug delivery to cells overexpressing the EGFR and EGFRvIII mutation.

## 1. Introduction

The genetic and cellular heterogeneities of almost all cancer types continue to confound the development of effective cancer chemotherapies. To help solve this issue, several studies have highlighted the potential of combined treatment with drugs differing in their mechanism of action [1,2,3,4], based on the rationale that such combinations will significantly reduce the probability of the outgrowth of clones resistant to both drugs. However, such resistance does occur and drug resistance is a major cause of therapeutic failure [5]. One example is the use of small molecules and antibodies to treat cancers overexpressing the epidermal growth factor receptor (EGFR) [6]. EGFR plays a critical role in the regulation of cell proliferation, differentiation, and migration [7]. Its overexpression is frequently found in a variety of human tumors of epithelial origin, including non-small cell lung cancer (NSCLC), breast, head and neck, gastric, colorectal, prostate, esophageal, bladder, renal, pancreatic, and ovarian cancers [8]. NSCLC is the most common form of lung cancer and is associated with a high morbidity and mortality rate characterized by a 5-year survival rate of only 11–15% [9]. EGFR overexpression has been identified in 40–89% of NSCLC cases. 

The EGFR gene can undergo several mutations, particularly within exons 18–21, which encode a portion of the EGFR kinase domain that has been associated with some types of lung cancer [10]. The most common variant is EGFR variant III (EGFRvIII) in which exons 2–7 are deleted [11,12,13]. The EGFRvIII mutation was suggested as a marker for a cancer stem cell or tumor-initiating population [11] and its presence is associated with more aggressive disease and poorer prognosis [12]. Two classes of EGFR inhibitors have been clinically approved, namely monoclonal antibodies (cetuximab, panitumumab), which target the extracellular domain of EGFR [14], and small molecule kinase inhibitors (gefitinib, erlotinib), which block the intracellular phosphorylation of the receptor [15]. Despite a good initial response rate to these drugs, most patients eventually develop drug resistance [16,17]. Hence, new treatment approaches are needed. 

One strategy that could effectively overcome the hurdle of drug resistance is to employ drug delivery systems that target overexpressed survival and proliferation-related receptors [3,18,19,20,21,22], utilizing them to specifically deliver cytotoxic drugs into cancer cells [23,24,25,26,27]. In this scenario, the use of S-S bridged cyclic peptides incorporated into peptide–drug conjugates (PDCs) carries a number of key advantages, as described elsewhere [28,29,30]. In particular, cyclic PDCs are conformationally more constrained structures with enhanced metabolic stability and binding specificity (to target molecules) compared to their linear counterparts. This leads to enhanced pharmacokinetics and effectiveness of the drug delivery [18,31]. 

The aim of this study was to discover new cyclic peptide ligands that selectively bind to both EGFR and EGFRvIII and evaluate the targeting cytotoxicity of their PDCs bearing the topoisomerase I inhibitor camptothecin (CPT) for NSCLC and glioblastoma cell lines. 

## 2. Methods

### 2.1. General

The LC/MS analysis was performed using an Agilent Technologies 1260 Infinity (LC) 6120 quadruple (MS), column Agilent SB-C18, 1.8 mm, 2.1 × 50 mm, column temperature 50 °C, eluent water–acetonitrile (ACN) + 0.1% formic acid. HRMS was performed in an ESI-positive mode by using an Agilent 6550 iFunnel Q-TOF LC/MS instrument. ^1^H NMR and ^13^C NMR spectra were measured in CD_3_OD at 300 K on a Bruker AvanceIII HD (^1^H 400 MHz and ^13^C 100 MHz) spectrometer and a BBO probe equipped with a Z gradient coil. IR spectra of the different peptides were measured with a Jasco FTIR 4700 instrument. The spectral resolution was set to 2 cm^−1^ and 16 scans were taken per measurement. Absorption spectra were recorded on a Jasco V-730 UV–Vis spectrophotometer. Flow cytometry was performed on a Beckman CytoFLEX analyzer, and the data were evaluated with FlowJo software. Fluorescence images were recorded at a magnification of ×200 with a Zeiss LSM700 confocal laser microscope. PDCs were purified by preparative HPLC on an ECOM preparative system, with dual UV detection. A Phenomenex Gemini^®^ 10 µm RP18 (250 × 21.2 mm) column was used. The column was kept at ambient temperature. Eluents A (0.1% TFA in water) and B (0.1% TFA in CH_3_CN) were used. A typical elution was a gradient from 100% A to 100% B over 35 min at a flow rate of 25 mL/min^−1^. Chemical reactions were monitored by TLC (Silica gel 60 F-254, Merck) and LC/MS.

### 2.2. Materials

All solutions were prepared with Milli-Q water (Millipore Corporation, Bradford, MA, USA). Tryptone, yeast extract, disodium phosphate (Na_2_HPO_4_), potassium dihydrogen phosphate (KH_2_PO_4_), 5-bromo-4-chloro-3-indolyl-β-*D*-galactopyranoside (Xgal), isopropyl-β-*D*-thio galactopyranoside (IPTG), 3-[(3-cholamidopropyl) dimethylammonio]-1-propanesulfonate (CHAPS), glycine, sodium dodecyl sulfate (SDS), Triton X-100, NaCl, polyethylene glycol (PEG) 8000, agarose, and glycerol were purchased from Alfa Aesar, Israel or Sigma-Aldrich, Israel. Epidermal growth factor (EGF) was purchased from Abcam (abcam, Waltham, MA, USA). The Ph.D.-C7C Phage Display Peptide Library kit containing *Escherichia coli* host strain ER2738, a robust F+ strain with rapid growth and M13 phage displaying 7 mer cyclic peptides, was from New England Biolabs, Ipswich, MA, USA. The Dulbecco’s Modified Eagle Medium (DMEM), RPMI-1640, trypsin, fetal bovine serum (FBS), XTT, penicillin, and streptomycin were obtained from Sartorius, Israel. Mouse antibody APC anti-human EGFR, APC Goat anti-mouse IgG, cell staining buffer, and DMSO were obtained from ENCO, Israel. Mouse monoclonal antibody anti-EGFRvIII mutation, clone L8A4, was obtained from Merck Millipore.

### 2.3. Cells and Culture Conditions

DKMG cells were purchased from the DSMZ cell bank (Braunschweig, Germany). Lung cancer (H1299, H1975), myeloid leukemia (K562), breast cancer (MDA-435), normal breast (MCF-10A), and embryonic (HEK-293) cell lines were obtained from the ATCC. All cell lines were cultured in RPMI 1640 or DMEM medium supplemented with 10% fetal bovine serum (FBS), 2 mM L-glutamine, and 1% penicillin/streptomycin (all from Sartorius, Israel). Cells were maintained at 37 °C in humidified 5% CO_2_ atmosphere. H1299, H1975, and DKMG cells were characterized by cell surface overexpression of EGFR [32]. DKMG cells are known to express two forms of EGFR—wild type (WT) and another carrying a mutation in the external region of the receptor (EGFR^vIII^) [33]. H1975 cells carry a mutation in the internal domain of EGFR (L858R/T790M) [34].

### 2.4. Positive In Vitro Biopanning Selection for Specific Peptides

The phage display kit was used for the in vitro biopanning experiments according to the manufacturer’s instructions. All cell lines were individually incubated with phages from the stock library. K562 cells, which do not express EGFR, were used for negative biopanning selection. Adherent cells (H1299, H1297, and DKMG) were each plated at a density of 10^5^ cells/mL in a 6-well plate. When the culture reached 80% confluence, one well was incubated with the phage library (10^11^ pfu/10 µL) for 1 h at 37 °C with gentle stirring. The medium containing the unbound phage was collected and transferred to a second well and incubated again. The procedure was repeated with the third well. Cells in the first and third wells were washed four times with 0.5% Tween-20 in PBS. Then, 0.5 mL of elution buffer (0.2 M glycine-HCl, pH 2.2) was added and incubated for 10 min at 4 °C; the pH was then neutralized by the addition of 75 µL of 1 M Tris–HCl buffer, pH 9.0. The supernatant containing the cell surface binding phage was collected. The cells were then lysed by incubation for 1 h at 4 °C with 2 mL of 30 mM Tris–HCl and 1 mM EDTA, pH 8.0, the medium was collected and centrifuged at 265× *g* for 5 min and the supernatant containing the cell-internalized phage was transferred to a new tube. The internalized and surface-bound phages were amplified according to the manufacturer’s instructions. An aliquot of the phages was retained for sequencing (see below) and the remainder was subjected to two additional rounds of biopanning. For K562 cells, the same scheme was used, except that the cells were washed by centrifugal pelleting for 5 min, RT, 265× *g*.

### 2.5. DNA Preparation and Sequencing

Following biopanning, DNA was extracted from first and third round phages as well as from the original phage pool, according to the manufacturer’s instructions. Libraries were prepared for NGS, and sequencing was performed by Hylabs Pty, Ltd. using MiSeq technology (Rehovot, Israel).

### 2.6. Analysis of NGS Data

For the analysis of DNA sequencing data, a specific Perl script was written. The workflow of the data processing included translation; generation of a report list sorted by the number of amino acid sequence repeats; and curation of the list by removal of sequences internalized by K562 cells or those that did not appear in the original Ph.D.-C7C Phage Display Peptide Library. From this workflow, 11 peptides were selected, from which, two sub-lists were generated: one list included those clones internalized by all 3 cell lines H1299, H1975, and DKMG; the second included a list of peptides internalized uniquely by each cell line.

### 2.7. Solid-Phase Synthesis of Peptides

Eleven 7-mer S-S bridged cyclic peptides were synthesized by solid-phase peptide synthesis on 2-chlorotrityl chloride resin (2-CTR, loading 1.0–1.5 mmol/g, 100–200 mesh, Sigma-Aldrich, Rehovot, Israel), as free peptides or conjugated to fluorescein isothiocyanate (FITC) or camptothecin (CPT) through γ-aminobutyric acid GABA linker using standard Fmoc protocols [35]. The cyclization between two cysteine amino acids of the peptides was carried out prior to the conjugation step using I_2_ (10 eq.) in DMF/H_2_O (4:1) for 2 h [36]. The purity of the peptides was no less than 95% (according to HPLC data measured at 254 nm, see Supplementary).

### 2.8. Peptide Conjugation to FITC and CPT

The deprotected cyclic peptides on the resin were coupled to the Fmoc-GABA-OH linker (2 eq.) for 2 h using benzotriazol-1-yloxytripyrrolidinophosphonium hexafluorophosphate (PyBOP) (2 eq.) as a coupling reagent and diisopropylethylamine (DIPEA), (6 eq.), as a base in DMF during the 2 h. Further, the Fmoc protecting group was removed (20% piperidine in DMF, 2 × 15 min), the resin was washed with DMF 3 × 5 min, DCM 3 × 5 min, followed by conjugation to FITC: FITC (2 eq.) in DIPEA (6 eq.) in NMP for 2 h. For CPT: 4-Nitrophenyl camptothecin-10-yl carbonate (CPT–4NFCF) [37] (2 eq.) in DIPEA (6 eq.), DIMAP (0.5 eq.) in DMF for 2 h. The cyclized PDCs were washed (DMF 3 × 5 min, DCM 3 × 5), dried (vacuum), and cleaved from the resin by treating with a cold TFA cocktail (95% TFA, 2.5% TIS, 2.5% H_2_O) during the 2 h. Then, the solvent was evaporated (N_2_) and the crude PDCs were purified by preparative HPLC on an ECOM preparative system, with dual UV detection. A Phenomenex Gemini^®^ 10 µm RP18 (250 × 21.2 mm) column was used. The column was kept at 25 °C. Eluents A (0.1 % TFA in water) and B (0.1% TFA in CH_3_CN) were used. A typical elution was a gradient from 100% A to 100% B over 35 min at a flow rate of 25 mL/min. For the P6 FITC conjugate: (22.6 mg, 22% total yield, purity 95%) LC-MS: RT = 8.66 min; ESI-HRMS m/z (M+) calc. 1449.4940 (C_66_H_77_N_15_O_17_S_3_), found 1449.5023. For the P6 CPT conjugate: (14.1 mg, 17% total yield, purity 96%); LC-MS: RT = 4.79 min; ESI-HRMS m/z (M+) calc. 1434.5612 (C_67_H_83_N_15_O_17_S_2_), found 1434.5562. For the P9 FITC conjugate: (20.7 mg, 19% total yield, purity 97%). LC-MS: RT = 7.94 min; ESI-HRMS m/z (M+) calc. 1489.4607 (C_65_H_80_N_14_O_19_S_4_), found 1489.4673. For the P9 CPT conjugate: (11.8 mg, 14% total yield, purity 95%). LC–MS: RT = 5.21 min; ESI-HRMS m/z (M+) calc.: 1474.5252 (C_65_H_83_N_15_O_19_S_3_) found: 1474.5281. 

### 2.9. Drug Release Profiles

PDC breakdown rates were determined as described elsewhere [35]. Briefly, peptide-FITC and peptide–CPT conjugates were incubated at 37 °C in 1 mL of DMEM or RPMI full-growth medium for 0, 0.6, 3, 7, 24, 48, and 72 h. At each time point, 10 µL of the sample was taken and mixed with 25 µL ACN/H_2_0 (1:1, *v*/*v*.). The samples were centrifuged at 1400 rpm for 15 min. Supernatants were collected, filtered, and analyzed by liquid chromatography–mass spectrometry (LC–MS). 

### 2.10. Detection of EGFR+ve/EGFRvIII Expression by Flow Cytometry

H1299 and DKMG cells were collected after treatment with trypsin, resuspended in Flow Cytometry Staining Buffer (FCSB, Sartorius, Israel), and centrifuged for 5 min 265× *g*. K562 suspension cells were directly resuspended in FCSB. A population of 10^6^ cells/mL was then incubated with mouse anti-human EGFRvIII primary followed by two washings with FCSB and incubation with APC anti-mouse Ig antibody at 4 °C for 20 min in the dark or directly stained with anti-human EGFR-APC antibodies at 4 °C for 1 h in the dark. After washing, cells were resuspended in 200 μL of FCSB and analyzed by flow cytometry. For each sample, 5 × 10^4^ cells were examined. 

### 2.11. Binding and Internalization Analysis of FITC-Conjugated Peptides

Cell cultures of 80–90% confluence were incubated with 2.5 µM FITC-conjugated peptides in RPMI–5% FBS for 1 h at 37 °C. Cells were scrapped from the culture flask, washed twice with 2 mL FCSB, and analyzed by flow cytometry to measure internalization. The cells were trypsinized after removal of the unbound conjugate, washed twice with FCSB, resuspended in 200 μL of FCSB, and analyzed by flow cytometry. 

### 2.12. Confocal Microscopy 

H1299, DKMG, and HEK-293 cells were seeded in 12-well glass bottom black plates in concentrations of 5.0 × 10^4^, 1.0 × 10^5^, and 2.5 × 10^4^ cells/well, respectively. The cells were cultured in a complete growth medium for 24 h. After incubation, the medium was removed, the cells were twice washed with PBS containing Ca^2+^Mg^2+^, and a fresh medium (0.5 mL) containing 25 µM of FITC labeled P6 or P9 peptide conjugate was added. The cells were incubated at 37 °C under 5% CO_2_ for 0 h and 3 h, washed twice with PBS and stained with Hoechst 33258 (16.23 mM, 0.5 mL, 37 °C, 5% CO_2_) for 10 min. The cells were washed twice with PBS and fixed with 4% paraformaldehyde.

### 2.13. Competitive Binding Assay

H1299 and DKMG cell lines were cultured in a complete RPMI growth medium in a 6-well plate overnight, until 80% confluent. Pre-incubation was performed with a blocking buffer consisting of 5 mg/mL BSA in PBS, pH 7.4, at 4 °C for 30 min. Then, cells were washed twice in PBS and incubated with 2.5 µM of FITC-labeled peptides with or without 80 nM EGF at 4 °C for 1 h. The cells were then incubated for 15 min at 37 °C under 5% CO_2_ to allow for peptide internalization. The cells were washed twice by centrifugation at 265× *g* for 5 min and analyzed by flow cytometry. 

### 2.14. Peptide Docking

The three-dimensional structures of the peptides were predicted using the PEPstrMOD [38,39] server. The EGFR structure (Protein Data Bank [40,41] code 1IVO [42]) was used for docking simulations of the peptides to the receptor. This structure represents the human EGFR extracellular region in a complex with EGF ligands. The ligands were removed from the structures prior to the docking simulations. Unbiased rigid body docking (exhaustive search of all possible binding sites and binding poses) was performed using three servers, HDOCK [43,44], LZerD [45,46], and ZDOCK [47]. Next, the best binding poses obtained from each of the three servers were optimized using the local docking protocol of the RosettaDock server [48,49], which identifies low-energy conformations by optimizing rigid-body orientations and side-chain conformations. The three best local docking poses of each optimization run (a total of nine) were minimized using UCSF Chimera [50] and the peptide-EGFR binding energy was assessed using PRODIGY [51,52].

### 2.15. Cellular Toxicity of the Peptide–CPT Conjugates 

Cell growth in the presence of PDCs was measured by a commercial XTT assay kit (Cell Proliferation Kit, XTT based; Sartorius, Israel). The cells were seeded in 96-well plates (10^4^ cells/well) and incubated overnight in a complete growth medium. They were then washed and re-cultured in 100 µL of fresh complete medium containing various concentrations of free drugs or PDCs, for 24, 48, and 72 h. The XTT solution was added to each well and the plate was further incubated for 2–3 h at 37 °C. The optical densities in the wells were measured at both 480 and 680 nm using a TECAN Infinite M200 ELISA reader. All tests were performed in triplicate, and each experiment was repeated three times. 

### 2.16. Statistical Analysis

All experiments were performed in triplicate and the results are expressed as the mean ± standard deviation. Data were analyzed using GraphPad Prism 6.0 (GraphPad Software, Inc., San Diego, CA, USA) [53]. Statistical analysis was performed by the two-way ANOVA test. The *p*-value < 0.05 indicated a statistically significant difference.

## 3. Results

### 3.1. Evaluation of Cell Surface Expression of EGFR^WT^ and EGFRvIII by Flow Cytometry

**Figure 1:**
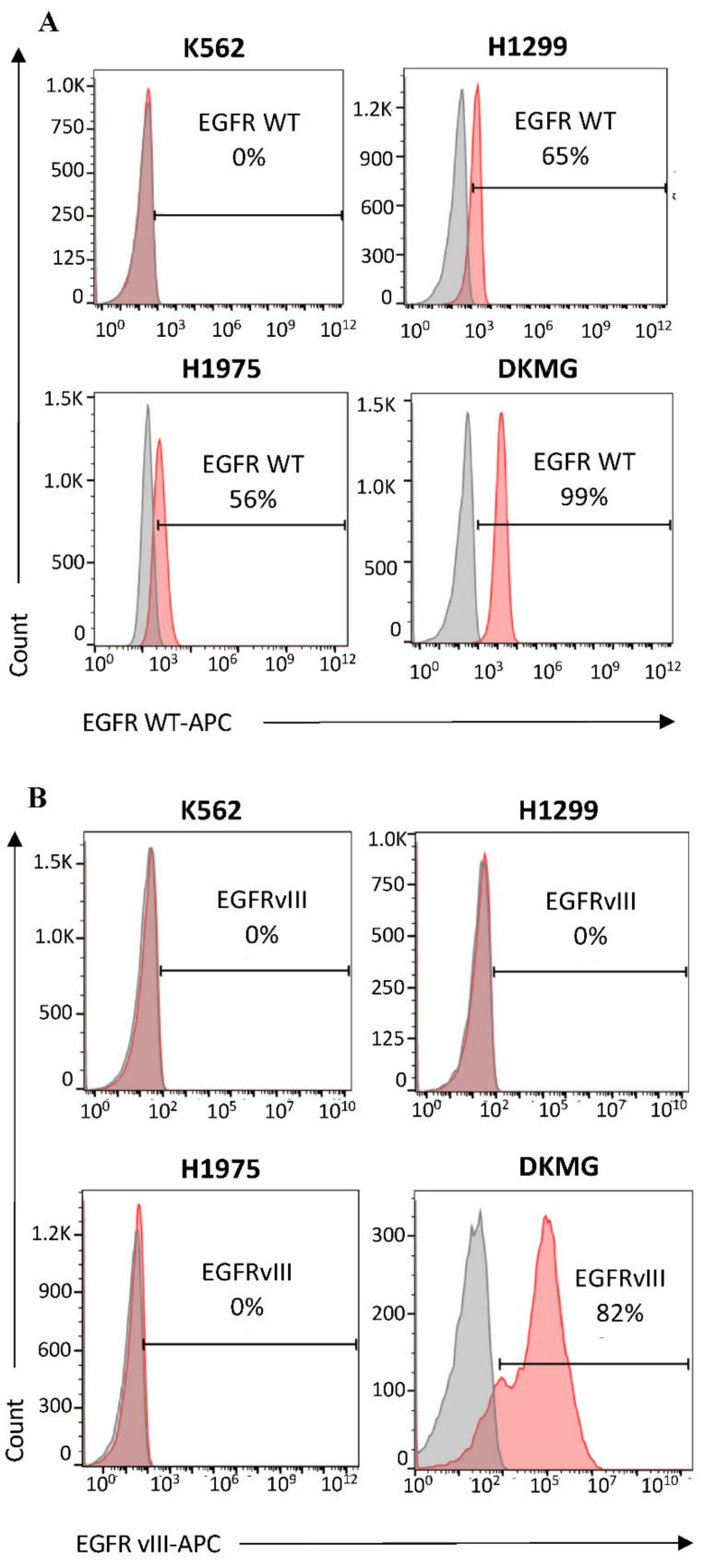
**Detection of EGFR^WT^ and EGFRvIII expression in K562, H1299, H1975, and DKMG cells by flow cytometry**. Cells were incubated with (**A**) anti-human EGFR WT-APC or (**B**) anti-human EGFRvIII antibodies for 1 h at 4 °C, and were washed and analyzed by flow cytometry. The gray curves are unstained cells, and the red curves indicate positive cells for EGFR^wt^ or EGFRvIII. For each sample, 20,000 cells were examined. The data shown are from one experiment that is representative of three repeated experiments.

Prior to using H1299, H1975, DKMG, and K562 cells in biopanning, the expressions of EGFR^WT^ and EGFRvIII were confirmed using flow cytometry. As expected, H1299, H1975, and DKMG cells expressed EGFR^WT^ (65%, 55%, and 92%, respectively) (Figure 1A), whereas only DKMG cells expressed EGFRvIII (82%) (Figure 1B). K562 cells did not express either of these receptors and were used as the negative control [54,55] (Figure 1A,B).

### 3.2. Selection of EGFR-Specific Peptides

To isolate peptides that specifically bind and internalize into EGFR+ve cells, the Ph.D.-C7C Phage Display Peptide Library was used. For each cell line, three rounds of biopanning were performed and both surface and internalized phages were recovered. DNA from phage pools was extracted from the first and the third rounds sequenced by NGS and translated.

To select the target-specific peptides, we first validated that all of the peptide sequences appeared in the original phage display library. Then, peptides derived from the EGFR−ve K562 cells were excluded from the list. After this step, 617,503 sequences remained. Finally, two groups of peptides were constructed. The first included sequences that were present in all three EGFR+ve cell lines; the second was divided into three subgroups, one for each cell line (Table S1). From this list, 11 sequences with the highest number of repeat reads were selected (Table S2).

### 3.3. Binding and Internalization of the Selected Peptides

The 11 selected peptides were synthesized together with a FITC tag. The formation of the thiourea group upon the FITC-to-peptide binding was confirmed by FTIR. In the FITC spectrum, there is a characteristic signal of the N=C=S group at 2015 cm^−1^ [56], which disappears upon conjugation with peptides indicating the formation of the PDCs (see Supplementary). Other fingerprint signals of both counterparts are present in the PDC spectra. The formed thiourea group of PDCs (anticipated location at 1585 cm^−1^ and 1449 cm^−1^ [57] cannot be recognized in the FTIR spectra because its signal is overlapped by numerous amide bands at 1470–1570 cm^–1^ [58].

**Figure 2:**
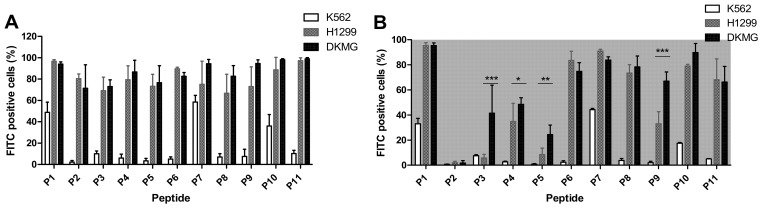
**Biological activity of the peptide–FITC conjugates**. (**A**) Binding and (**B**) internalization activity of P1-P11 FITC-labeled peptides on K562, H1299, and DKMG cells measured by flow cytometry. Cells were treated with 2.5 µM peptide–FITC conjugates and incubation for 3 h at 37 °C before the FACS analysis. The experiments were repeated three times. Mean and ±SD are shown (* *p* < 0.05, ** *p* < 0.01, *** *p* < 0.001).

The binding and internalization capabilities of the obtained conjugates were tested at 2.5 µM on K562, H1299, and DKMG cells. This concentration was chosen based on a preliminary dose-dependence study, which showed receptor saturation from 5 µM (Figure S1). The total number of cells that bound but did not internalize peptides (Figure 2A) was calculated by subtracting the number of peptide-internalized cells (Figure 2B) from the total number of positive cells. The flow cytometry analysis showed that FITC-labeled peptides P2–P6, P8, P9, and P11 peptides selectively bind to H1299 and DKMG but not K562 cells. Furthermore, the FITC-labeled P3, P6, P8, P9, and P11 peptides internalized into EGFR+ve cells (Figure 2B). FITC-labeled P3–P5 and P9 showed higher internalization activity to the DKMG cell line expressing both EGFR+ve and EGFRvIII mutations.

### 3.4. Competitive Binding of Peptides to EGFR

**Figure 3:**
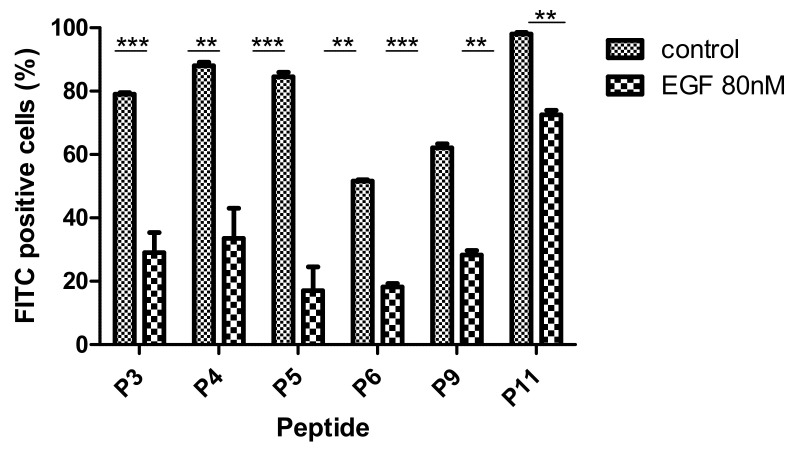
**Competitive uptake of P3, P4, P5, P6, P9, and P11 peptides by FACS.** Fluorescence was measured for H1299 cells (30,000 cells) after incubation with (2.5 µM) P3–P6, P9, and P11 by FITC-labeled peptides in the absence or presence of 80 nM of the EGF protein. The experiments were performed in duplicate. For each sample, the mean ± SD is shown (** *p* < 0.01, *** *p* < 0.001).

To validate whether the seven internalized peptides target the EGFR receptor, a competitive binding test with the natural ligand EGF was performed. Figure 3 shows that EGF significantly competed with the peptides P3–P6, P9, and P11. As EGFRvIII is activated independently of EGF stimulation [59,60], no competitive test was done on the DKMG EGFRvIII expressing cells.

### 3.5. Evaluation of Target Specificity of the Peptides towards EGFR Expressed Cells by Flow Cytometry

**Figure 4:**
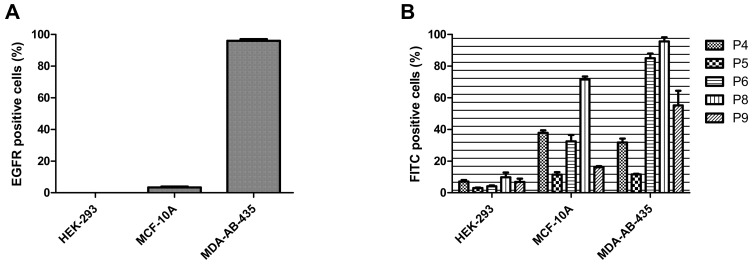
**EGFR status and peptide internalization into HEK-293, MCF-10A, and MDA-MB-435 cells.** (**A**) Flow cytometry analysis of EGFR expression in cells. Cells were treated with monoclonal antibodies against human EGFR. Expression levels were measured by the APC fluorescence intensity. (**B**) Cells were incubated with 2.5 µM peptides for 3 h at 37 °C, collected with trypsin, and analyzed by flow cytometry. The experiment was repeated three times; untreated cells were used as the control. The mean of the FITC positive cells ± SD is presented (n = 5).

Target specificities of the FITC-labeled P4–P6, P8, and P9 peptides were investigated on three cell lines, which were not used in biopanning: kidney HEK-293, normal breast MCF-10A cells, and MDA-MB-435 breast cancer cells. To verify EGFR^WT^ expression on these cell lines, they were stained with an APC-labeled monoclonal anti-human EGFR antibody followed by a flow cytometry analysis. EGFR^WT^ expression on HEK-293 and MCF-10A cells was negligible (less than 10%), while about 97% of MDA-MB-435 cells were EGFR^WT^-positive (Figure 4A).

The interactions of the FITC labeled P4–P6, P8, and P9 with these cell lines were then analyzed by flow cytometry. All peptides showed weak binding to EGFR-negative HEK-293 cells, while P6 and P9 showed a pronounced into high-EGFR-expressing MDA-MB-435 cells; P8 was less EGFR-specific, exhibiting appreciable binding to both MCF-10A and MDA-MB-435 cell lines (Figure 4B). These results indicated that the most target-specific peptides were P6 and P9.

### 3.6. Evaluation of Target Specificity of the Peptides towards EGFR-Expressed Cells by Fluorescence Microscopy

**Figure 5:**
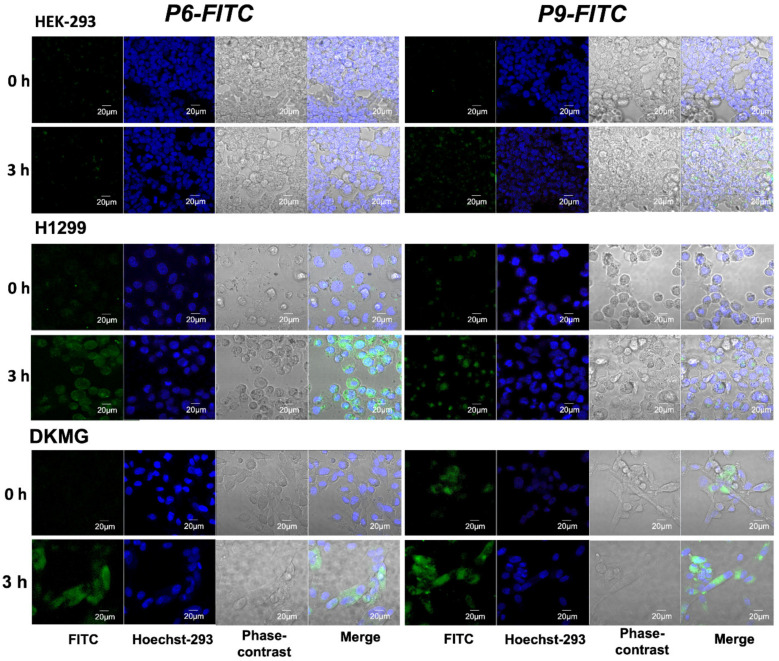
**Intracellular localization of FITC-labeled P6 and P9 peptides. HEK-293, H1299, and DKMG**. Cells were grown in glass-bottom black plates and incubated for 0 and 3 h at 37 °C with 25 µM of FITC-labeled P6 and P9 peptides. Cell nuclei were stained with Hoechst-293. After incubation, cells were washed three times with PBS (Ca^2+^ and Mg^2+^), fixed with 4% paraformaldehyde (PFA), and then analyzed by confocal laser microscopy. Magnification ×200.

To further the applicability of P6 and P9 to target EGFR+ve cancer cells, confocal microscopy was used to observe peptide internalization into EGFR^WT^ H1299 lung cancer cells, EGFRvIII DKMG glioblastoma cells, and EGFR−ve HEK-293 normal cells (Figure 5). The cells were incubated with 2.5 μM FITC-labeled P6 and P9, their nuclei were stained with Hoechst-293, the cells were fixed with 4% paraformaldehyde, and photographed immediately and after 3 h of incubation. Importantly, all the cells exhibited the blue fluorescence signal of Hoechst-293, which evidenced their viability. There was a clear correlation between the FITC signal strength and EGFR expression. After 3 h, FITC-P6-strongly stained H1299 and DKMG cells, but not HEK-293 cells. In contrast, FITC-P9 only exhibited strong staining with DKMG cells.

### 3.7. Peptide Docking

**Figure 6:**
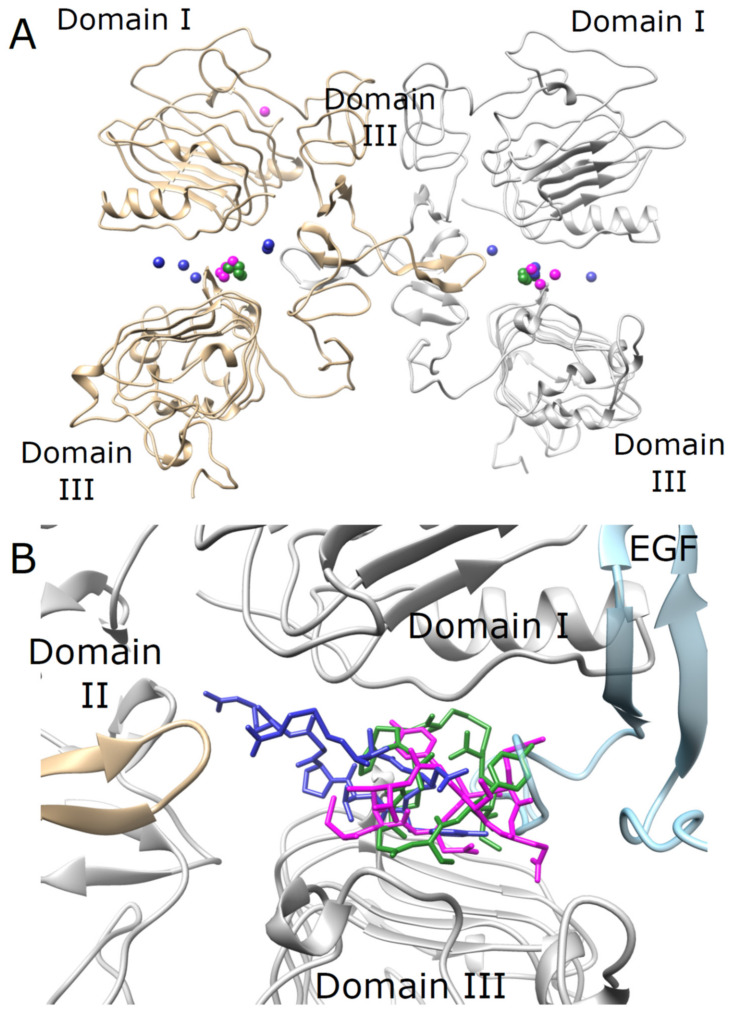
**Docked structures of peptides P6, P9, and P11 to the EGFR extracellular region.** (**A**) Unbiased rigid body docking of the peptides to EGFR was carried out with the HDOCK server. The top ten docked poses of each peptide are represented as spheres corresponding to their centers of mass, and colored green (P6), magenta (P9), and blue (P11). The homodimeric structure of EGFR is shown using ribbon representation, with chains A and B colored gray and gold, respectively. The vast majority of peptides were bound to the EGFR receptor in the cavity between domains I and III. (**B**) The docked structure of peptides P6 (green), P9 (magenta), and P11 (blue) are shown using a stick representation. The peptides were bound to the EGFR receptor in the cavity between domains I and III in vicinity to the EGF binding site. Peptides P6 and P9 show substantial overlap with EGF while peptide P11 is bound deeper within the cavity (toward domain II) and has a smaller overlap with EGF. For clarity, the EGF structure (cyan) is shown using semitransparent ribbon representation and removed from the structure during the docking simulations.

Unbiased rigid body docking simulations were carried out between the peptides and the EGFR receptor to identify their binding sites within the receptor. The best ten docking results obtained from the HDOCK server [43,44] for each peptide are shown in Figure 6A. Each peptide is presented using a sphere corresponding to its center of mass and colored green (P6), magenta (P9), and blue (P11). Most of the docked structures were bound to the receptor in the cavity between domains I and III. Similar results were obtained with the LZerD [45,46] and ZDOCK [47] docking servers (see Supplementary Figure S2). After identification of the binding site, optimization of the binding pose was carried out using the RosettaDock server [48,49]. The best-docked pose of each peptide from each server underwent a local docking protocol that enabled side chain movements. The best three obtained locally docked structures (of each peptide) were then minimized and the binding energy of the peptide to the receptor was assessed using PRODIGY [51,52]. The best-docked pose of each peptide (lowest PROGIDY binding energy) is presented in Figure 6B. The peptides were bound to the EGFR receptor in the vicinity of the EGF binding site. P6 (green) and P9 (magenta) showed substantial overlap with EGF while P11 (blue) was bound deeper within the cavity (toward domain II) and had a smaller overlap with EGF. The PROGIDY-binding energies are −13.0, −11.9, and −11.3 kcal mol^−1^ for peptides P6, P9, and P11, respectively.

### 3.8. Synthesis of Peptide CPT Conjugates

**Figure 7:**
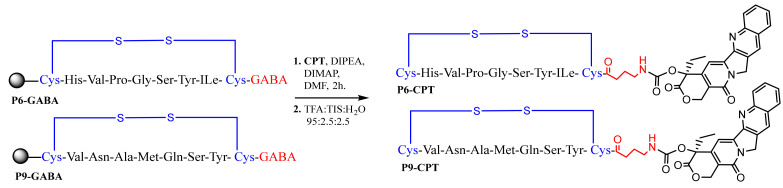
Synthesis of the **P6-CPT** and **P9-CPT** conjugates.

P6 and P9 were selected for further study and conjugation to camptothecin (CTP) (Figure 7). Notably, in its free form, CPT has high cytotoxic potency; however, it is poorly soluble in aqueous solution and produces off-target cytotoxicity, factors that have precluded its clinical utility. We hypothesized that conjugation of CPT to a targeting peptide would improve its pharmacological and targeting properties. On-resin intermediates P6 and P9 with deprotected GABA N-terminus were obtained after Fmoc removal of their precursors (20% piperidine in NMP, 10 mL). Thus, the free *N*-terminus amine of the resulting P6 and P9 were loaded with activated CPT, leading to the creation of biodegradable carbamate linkage. Finally, all the on-resin synthesized conjugates were cleaved from the solid support and precipitated by the addition of cold diethyl ether, isolated, lyophilized, and identified by LC–MS and HRMS, as peptide conjugates P6-CTP and P9-CPT.

For the CPT–peptide conjugates, the formed carbonate bond (1694 cm^−1^ and 1610 cm^−1^ in the FTIR spectra [61] is overlapped by numerous amide bonds at 1600−1700 cm^−1^ [58]. The disappearance of the hydroxyl group of CPT at around 3000 cm^–1^ upon the conjugation could not be detected because of the existence of the C-terminus carboxylic group of the peptides formed upon the cleavage from the Cl-Trt resin.

### 3.9. CPT Release from the Conjugates

**Figure 8:**
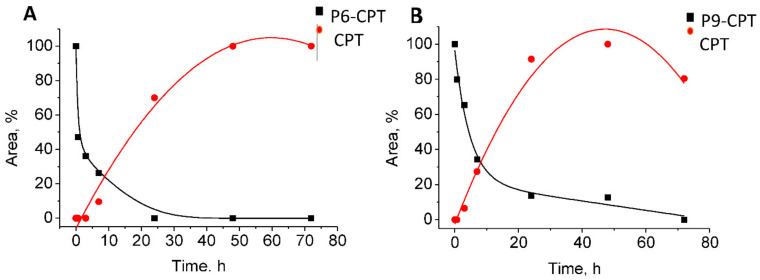
**CPT release profiles for P6-CPT and P9-CPT peptide drug conjugates**. Peptide conjugates were incubated at 37 °C in a complete growth RPMI medium for 0.5, 3, 7, 24, 48, and 72 h. (**A**) peptide 6 (**B**) peptide 9. The integrity of the conjugates was measured by mass spectrometry. Each measurement was performed in triplicate.

The release rates of CPT from P6-CPT and P9-CPT were measured in RPMI and DMEM complete growth media at 37 °C for 0, 0.5, 3, 7, 24, 48, and 72 h according to the procedure [35]. The degradation of the conjugates and the CPT release were analyzed by LC–MS. The CPT release rates for P6-CPT and P9-CPT were found to be similar. The conjugates degraded with a half-life of 6–7 h (Figure 8).

### 3.10. Effect of P6-CPT and P9-CPT PDCs on Cell Viability

The cytotoxicity of the PDCs was tested against H1299 and DKMG cells using two protocols. In the first protocol, cells were incubated in a complete medium containing a range of concentrations (0.5–50 µM) of P6, P9, P6-CPT, or P9-CPT for 6 h, after which, the medium was replaced with fresh, drug-free, complete medium, and the cells were cultured for an additional 24 h (designated as 6h24h) or 48 h (6h48h). In the second protocol, cells were exposed continuously to free drugs or PDCs for 24, 48, and 72 h. For each case, during the culture period, cell cytotoxicity was determined as described above and IC_50_ values were calculated. The results are presented in Table 1.

Overall, CPT did not result in pronounced cytotoxicity with the first protocol; however, the second protocol (continuous exposure) resulted in lower IC50 values as exposure time increased.

**Figure 9:**
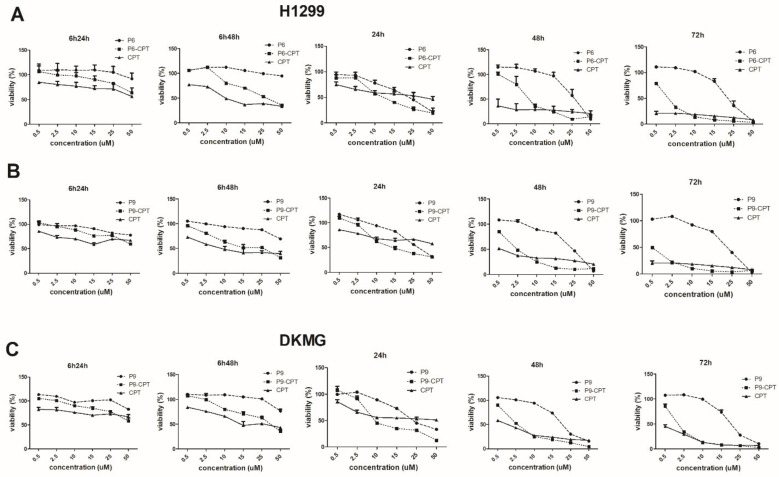
**Analysis of cytotoxicity of the P6, P6-CPT, P9, P9-CPT, and CPT.** (**A**) H1299 P6, (**B**) H1299 P9, and (**C**) DKMG P9 cells were cultured in a 96-well plate with six different concentrations of peptides, peptide conjugates, or CPT for 24, 48, and 72 h in two different protocols. The cell viability was measured by the XTT assay. Viability of the control non-treated cells was used as 100%. The viability of treated cells was represented as percentages of the control. Data are shown as Mean ± SD.

For H1299 cells, P6 and P9 peptides alone were not toxic even after 48 h of preincubation (Figure 9) while P6-CPT and P9-CPT exhibited cytotoxic effects after 48 h, although the IC_50_ was higher than for CPT alone (Table 1). However, with continuous exposure, P6-CPT and P9-CPT were cytotoxic after 24 h post-exposure (Figure 9). The IC_50_ of the PDCs was 2–3-fold lower compared to free CPT (Table 1).

For DKMG cells, a 24 h pre-incubation with P9 and P9-CPT did not affect cell viability. The cytotoxic effect of P9-CPT on DKMG was noted after 48 h while its IC_50_ concentration was four-fold higher compared to the free CPTs alone (Table 1). After continuous incubation for 24 h, the P9-CPT conjugate was more toxic (IC_50_ = 6.76 µM) compared to the free CPT (IC_50_ = 25 µM). After 48 and 72 h of incubation, P9-CPT demonstrated a similar cytotoxic effect as the free CPT. The cytotoxic effect of the peptide CPT conjugate was not tested on HEK normal cells, as the peptide did not bind or internalize into these EGFR-ve cells.

## 4. Discussion

In the present study, phage display technology and biopanning on viable cells were used to discover novel EGFR-specific cyclic peptides that could act as drug carriers in a targeted drug delivery system. For peptide selection, we utilized viable cells instead of purified target proteins. It might be suggested that the use of an isolated biomarker is a more direct approach to isolating a specific ligand from a library, as irrelevant molecules would not obscure the receptor of interest; however, it is difficult to isolate membrane proteins in a form that retains their natural configuration. Moreover, the use of whole cells allows for a more natural expression of cell surface receptors [62]. Validation of peptide specificity was performed by the use of non-EGFR expressing cells, competitive binding versus natural EGF, and simulated docking experiments.

Phage display biopanning was used previously to identify EGFR-binding peptides. For example, 17 peptides were selected for tumor imaging and targeted chemo- or photodynamic therapy in a murine model of colorectal cancer [63,64]. Other peptides were used to target murine neural stem cells for nerve regeneration [65], human hepatoma cells, and human chronic myeloid leukemia cells [66]. However, these peptides are linear and, therefore, suffer from several disadvantages, including low binding selectivity and low affinity, unstable three-dimensional structures, and susceptibility to extracellular protease degradation [18]. To circumvent these limitations, we chose to use a phage library displaying cyclic peptides.

When working with phage display technology, it is possible that a target receptor may bind a displayed peptide in associated with structures in the phage itself. Therefore, it is always important to synthesize the peptides displayed by selected candidate phage clones and then validate the target specificity of the free peptides. Indeed, we found that only 7 peptides of 11 originally selected candidates for target cell binding had the ability to be taken up by the cells and were therefore selected for further study. Four of these peptides showed high internalization into DKMG cells carrying the EGFRvIII mutation [63,64].

We did not directly investigate the mechanism(s) of the internalization of the selected peptides. However, peptide in silico docking studies with three peptides, P6, P9, and P11, confirmed their target specificity and showed binding at the active site of the EGFR. These three peptides showed good binding energy that ranged from −13.0 to −11.3 kcal mol^−1^. P6 and P9 showed higher steric clashes with EGF than P11. These docking results are in good agreement with the competitive uptake experiment (Figure 5), where these two peptides showed better competition with EGF compared to P11. These results strongly suggest that the peptides utilize EGFR receptors to gain entry into the cell.

Since the natural role of EGF is to stimulate intracellular signaling pathways important for cell proliferation [67], it is important to select peptides that specifically deliver drugs into the target cell, but do not stimulate proliferation. For this reason, P6 and P9 were selected for additional studies as drug carriers for the selective elimination of the EGFR expression tumor (Figure 8).

The cytotoxicity effects of PDCs depend on several factors, including biostability, the rate of penetration, efficiency of the drug release, and drug toxicity. The ability of the peptide–drug conjugates to remain stable and to specifically enter the target cells is an important factor in the delivery of highly toxic drugs [68,69]. Our PDCs showed half-lives of 6–7 h, which might be significantly enhanced by further manipulation, such as PEGylation [70], or conjugation to gold nanoparticles [25].

As found in previous studies, [25,71], conjugation of drugs to a targeting peptide can increase the IC50 value, as seen in the present study, with lower effectiveness of P6-CPT and P9-CPT compared to CPT alone after 6 h of preincubation with PDC. This is probably due to insufficient uptake of the drug and will depend, in part, on the extent of the receptor expression. While this higher IC_50_ would require an increase in the PDC dose, this would be offset by the enhanced target specificity and consequent decrease in off-target toxicity. An additional approach would be to arm the peptide with additional copies of the drug, as we have described elsewhere [22].

One of the features of glioblastoma (GBM) is the overexpression of EGFR [72] and its most common mutation EGFRvIII [73]. Patients with GBM respond very poorly to first- and second-generation EGFR inhibitors [74,75]. In this study, we found that peptide 9 binds specifically to both the WT and vIII mutant forms of EGFR and might therefore be a candidate carrier for targeted drug therapy in GBM. Expression of the EGFRvIII mutation has been noted in several other cancers as well [76] and has been suggested as a marker for cancer stem cells or a tumor-initiating population associated with more aggressive disease and poorer prognosis [77]. Further studies will test whether peptide 9 is suitable as a drug carrier for these targets.

## 5. Conclusions

In this work, we discovered a series of cyclic nonapeptides specific to EGFR-overexpressed H1299 and its mutation EGFRvIII-expressed DKMG cell lines using a combined phage-display cell-based positive biopanning selection method, followed by NGS sequencing of the collected DNA phages.

Apparently, two of these peptide ligands, P6 and P9. exhibited an effective binding and internalization to EGFR and EGFRvIII cell lines, as confirmed using FACS and fluorescent microscopy. Moreover, P6 and P9 conjugated to CPT enabled the nuclear accumulation of cytotoxic CPT. CPT intracellular accumulation resulted in DNA damage and induction of cell death. Hence, coupling of CPT to these peptides did not interfere with CPT cytotoxic effects, while contributing a specific targeting tool to EGFR and EGFRvIII overexpressed cancer cell lines. Docking simulations revealed that the peptides interact with the EGFR receptor in the vicinity of the EGF binding site, showing substantial overlap with EGF. We assume that P6 and P9 are promising peptide carriers for drug delivery to EGFR overexpressed cancers and their EGFRvIII mutation.

Further investigations of the therapeutic efficacies of PDCs based on these peptide carriers in various preclinical models are undergoing.

## Figures and Tables

**Table 1:** IC_50_ values (µM) of P6, P6-CPT, P9, and P9-CPT on H1299, DKMG cells. IC_50_ values were calculated using non-linear regression.

	H1299						DKMG	
***Incubation***	**P6**	**P6-CPT**	**P9**	**P9-CPT**	**CPT**	**P9**	**P9-CPT**	**CPT**
***6h24h***	>50% *	>50%*	>50% *	>50% *	>50% *	>50% *	>50% *	>50% *
***6h48h***	>50% *	32 ± 0.9	>50%	21.5 ± 2.1	6.7 ± 1.8	>50%	32 ± 1.6	8.5 ± 1.0
***24 h***	27.5 ± 0.8	14.5 ± 2.2	22.3 ± 0.2	8.4 ± 0.5	54.8 ± 2	16.4 ± 1.9	6.76 ± 1.4	25 ± 1.3
***48 h***	32 ± 2.8	5.5 ± 0.7	27.3 ± 1.7	2.2 ± 0.3	<45%	17.5 ± 2.3	2.7 ± 1.0	<50% **
***72 h***	28.8 ± 1.4	<27%	22.5 ± 0.0	1.2 ± 0.1	<15%	17.5 ± 1.6	0.2 ± 0.1	<40% **

* The cell viability was higher than 50% of the control untreated cells and IC_50_ could not be calculated. ** The cell viability was lower than 50% of the control untreated cells and IC_50_ could not be calculated.

## Data Availability

Not applicable.

## Supplementary Materials

The following supporting information can be downloaded at: https://www.mdpi.com/article/10.3390/pharmaceutics14071505/s1, Table S1. Selected C-terminus carboxylate cyclic peptide sequences. Table S2. The numbers of sequences after removing nonspecific peptides that were found in non-EGFR expressed K562 cells. Figure S1. Peptide’s titration and time-depended internalization assay. Figure S2. Unbiased rigid body docking of peptides P6, P9, and P11 to the EGFR extracellular region.
