# Supplementary material for: Novel Cyclic Peptides for Targeting EGFR and EGRvIII Mutation for Drug Delivery
Source: Pharmaceutics. 2022 Jul 20;14(7):1505. doi: 10.3390/pharmaceutics14071505 (PMC9318536; doi:10.3390/pharmaceutics14071505)
Supplement: Supplementary file 1 [file pharmaceutics-14-01505-s001.zip › pharmaceutics-1783823-SM.pdf]

Supplementary Information

Novel Cyclic Peptides for Targeting EGFR and EGRvIII Mutation for Drug Delivery

Olga Furman [1,2], Alisa Zaporozhets [3], Dror Tobi [4,5], Andrii Bazylevich [3], Michael A. Firer [1,4,6], Leonid Patsenker [3], Gary Gellerman [3,6] and Bat Chen R. Lubin [1,2,*,†]

[1] Department of Chemical Engineering, Biotechnology and Materials, Ariel University, Ariel 40700, Israel; olgaf@ariel.ac.il (O.F.); firer@ariel.ac.il (M.A.F.)
[2] Agriculture and Oenology Department, Eastern Regional R&D Center, Ariel 40700, Israel
[3] Department of Chemical Sciences, Ariel University, Ariel 40700, Israel; aliska.zaporozhets@gmail.com (A.Z.); andriib@ariel.ac.il (A.B.); leonidpa@ariel.ac.il (L.P.); garyg@ariel.ac.il (G.G.)
[4] Adelson School of Medicine, Ariel University, Ariel 40700, Israel; drorto@ariel.ac.il
[5] Department of Molecular Biology, Ariel University, Ariel 40700, Israel
[6] Ariel Center for Applied Cancer Research, Ariel 40700, Israel
* Correspondence: batchenl@ariel.ac.il; Tel.: +972-50-6554655
† Current address: Eastern Regional R&D Center, Room MOP 2.4, Ariel University, Ariel 40700, Israel.

Table S1. Selected C-terminus carboxylate cyclic peptide sequences. .

Peptide	Sequence	Cell lines
P1	c(CLRWRFGRC)	H1299, DKMG, H1975
P2	c(CVRWRFGRC)	
P3	c(CLAVEVRPC)	
P4	c(CSAETVESC)	
P5	c(CPNDSYHQC)	H1299
P6	c(CHVPGSYIC)	H1299
P7	c(CWHSLSLAC)	H1975
P8	c(CSALWASHC)	H1975
P9	c(CVNAMQSYC)	DKMG
P10	c(CNWLSRTEC)	DKMG
P11	c(CAQYTPGRC)	DKMG

Table S2. The numbers of sequences after removing nonspecific peptides that were found in non-EGFR expressed K562 cells.

#	Cell line	Number of common sequences for all 3 cell lines	Number of unique sequences for each line
1	H1299	416	1666
2	H1975		10437
3	DKMG		981

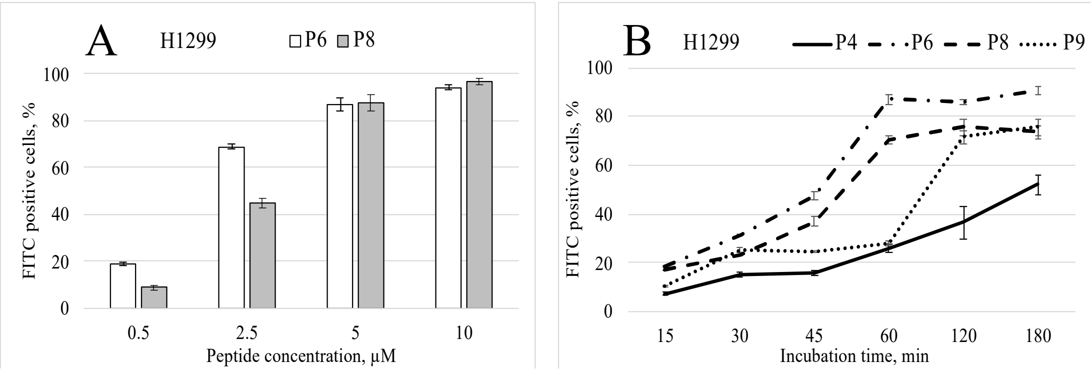

Figure S1. Peptide's titration and time-depended internalization assay.(**A**) H1299 cells were incubated during 1 h with 0.5 µM, 2.5 µM, 5 µM and 10 µM of FITC-labeled P6 and P8 peptides. **(B)** H1299 cells were incubated with 2.5 µM of FITC labeled peptides P4, P6, P8, and P9 for 15, 30, 45,60, 120 and 180 min. Cell analysis was performed using the flow cytometry. Mean ± standard deviation of 2 independent experiments in duplicates is shown.

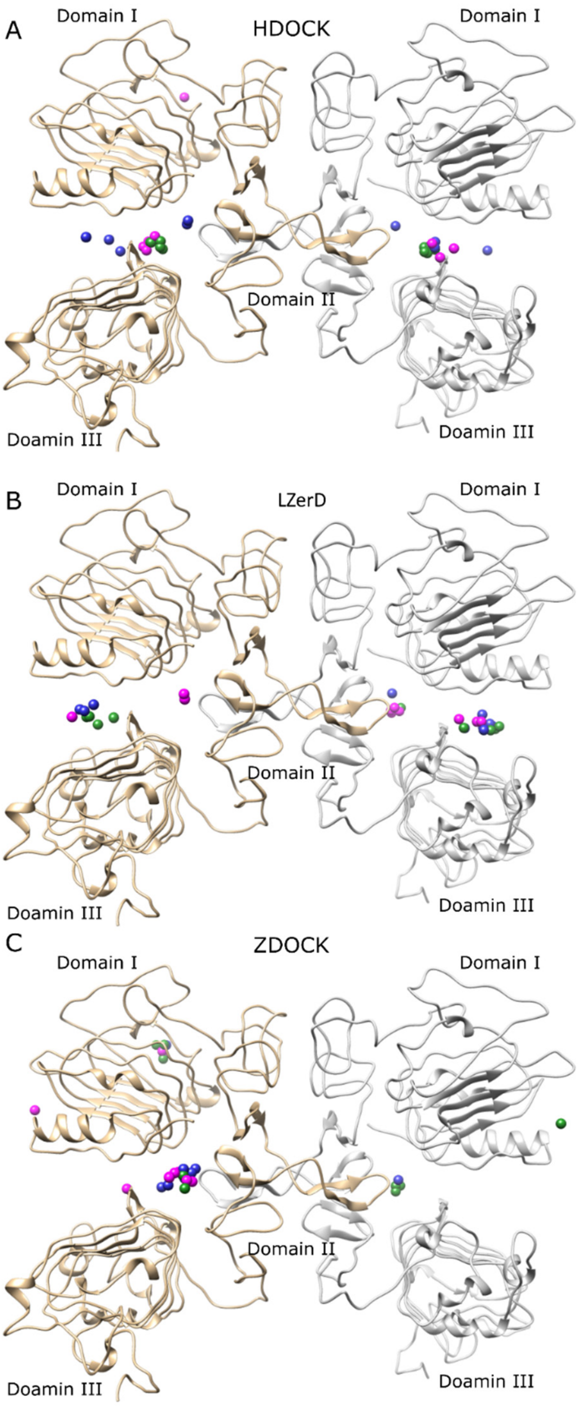

Figure S2. Unbiased rigid body docking of peptides P6, P9, and P11 to the EGFR extracellular region. Unbiased rigid body docking of the peptides to EGFR was carried out with (**A**) HDOCK (**B**) LZerD and (**C**) ZDOCK servers.

The top ten docked poses of each peptides are represented as spheres corresponding to their center of mass and colored green (peptide P6), magenta (peptide P9), and blue (peptide P11). The homodimeric structure of EGFR is shown using ribbon representation with chains A and B colored gray and gold, respectively. The results are similar for all three servers. Most of peptides are bound to the EGFR receptor in the cavity between domains I and III.

HRMS of P6-FITC

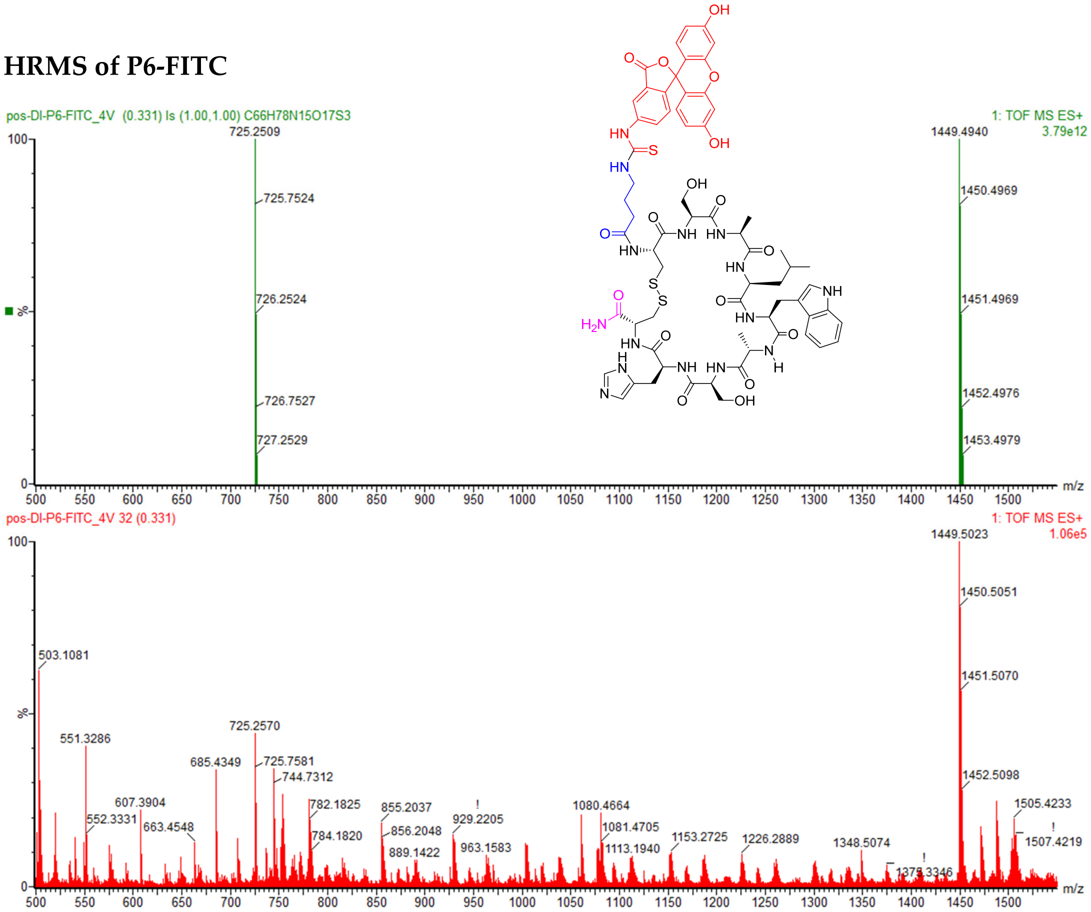

HRMS of P6-CPT

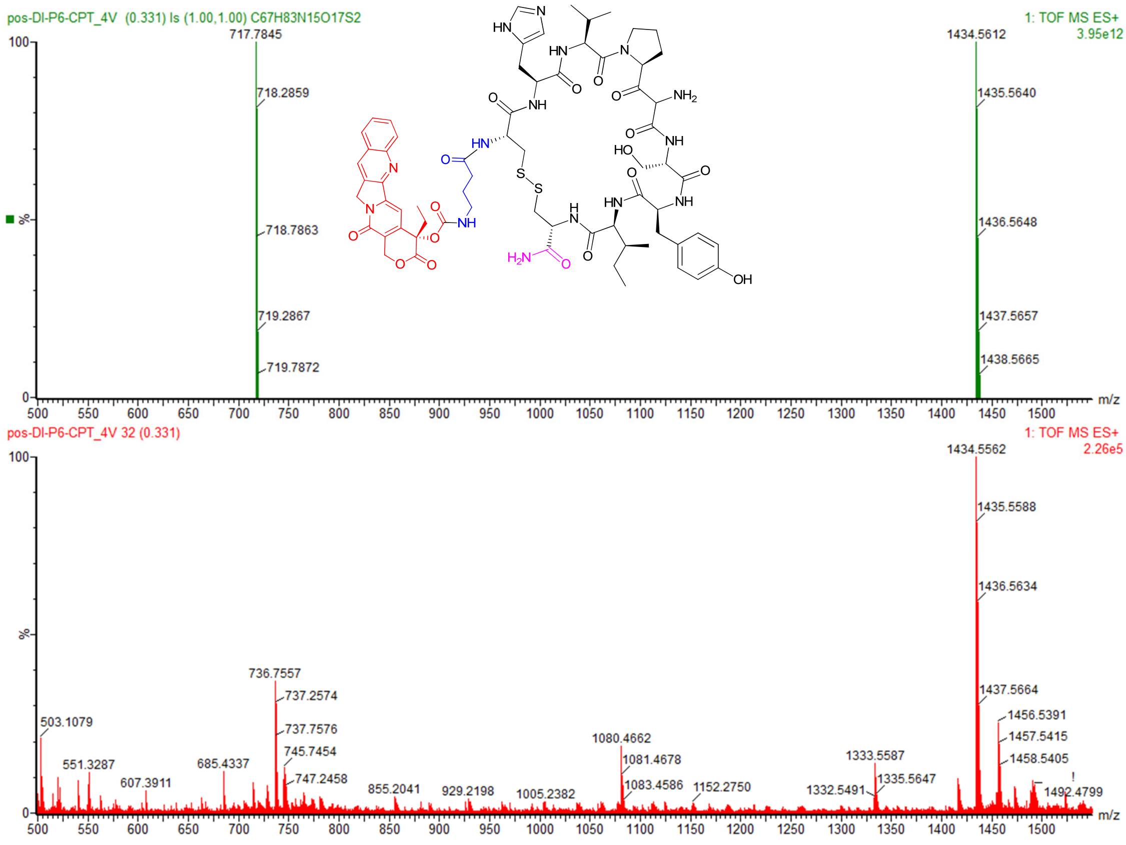

HRMS od P11-CPT

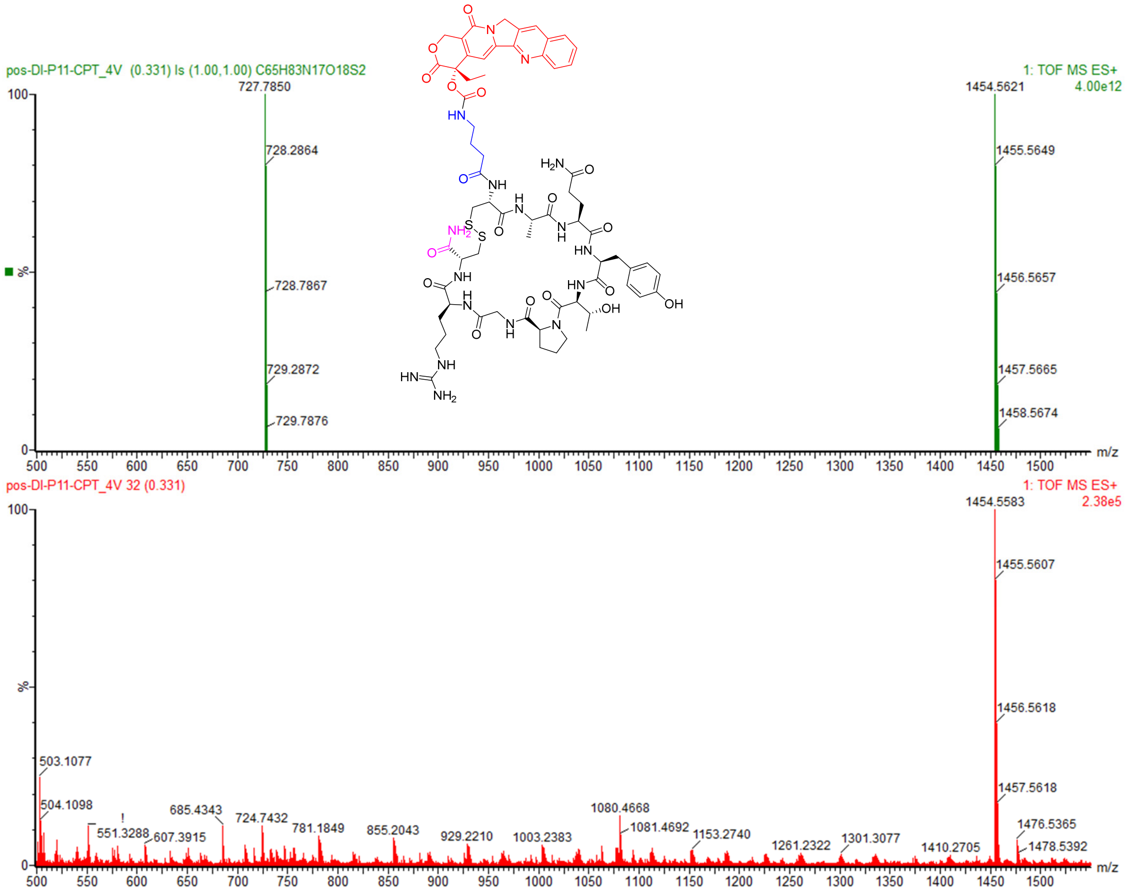

FTIR DATA of the peptide conjugates

P1 FITC

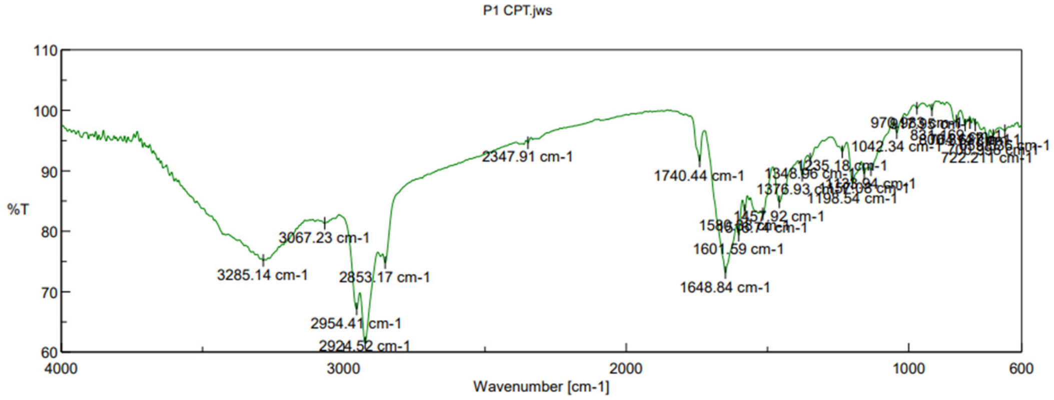

[Comments]		[Measurement Information]	
Sample name		Model Name	FT/IR-4700typeA
Comment		Serial Number	F061461788
User			
Division		Measurement Date	28/06/2022 12:13
Company	Ariel University		
		Light Source	Standard
		Detector	TGS
		Accumulation	70
[Detailed Information]		Resolution	4 cm-1
Creation date	28/06/2022 12:14	Zero Filling	On
		Apodization	Cosine
Data array type	Linear data array	Gain	Auto (8)
Horizontal axis	Wavenumber [cm-1]	Aperture	Auto (7.1 mm)
Vertical axis	%T	Scanning Speed	Auto (2 mm/sec)
Start	599.753 cm-1	Filter	Auto (30000 Hz)
End	4000.6 cm-1		
Data interval	0.964233 cm-1		
Data points	3528		

P1 CPT.jws

P11 CPT

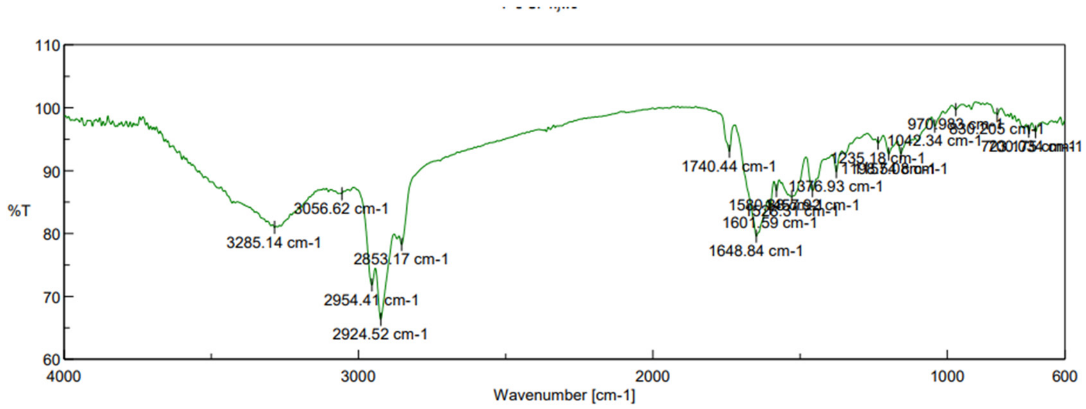

[Comments]		[Measurement Information]	
Sample name		Model Name	FT/IR-4700typeA
Comment		Serial Number	F061461788
User			
Division		Measurement Date	28/06/2022 12:01
Company	Ariel University		
		Light Source	Standard
		Detector	TGS
		Accumulation	50
[Detailed Information]		Resolution	4 cm-1
Creation date	28/06/2022 12:04	Zero Filling	On
		Apodization	Cosine
Data array type	Linear data array	Gain	Auto (8)
Horizontal axis	Wavenumber [cm-1]	Aperture	Auto (7.1 mm)
Vertical axis	%T	Scanning Speed	Auto (2 mm/sec)
Start	599.753 cm-1	Filter	Auto (30000 Hz)
End	4000.6 cm-1		
Data interval	0.964233 cm-1		
Data points	3528		

P 11 CPT.jws

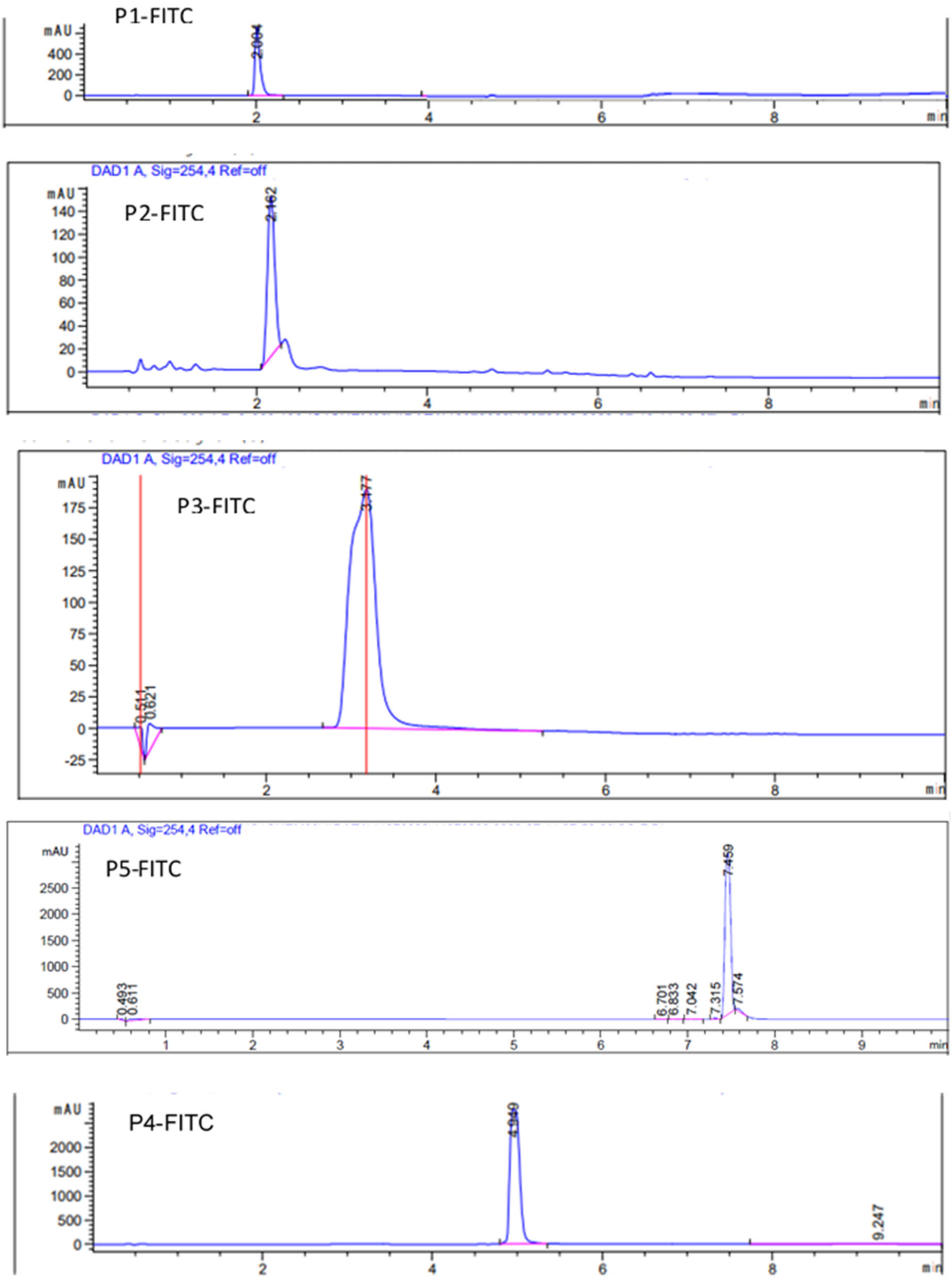
P1-FITC
mAU
min
DAD1 A, Sig=254,4 Ref=off
P2-FITC
2.162
DAD1 A, Sig=254,4 Ref=off
P3-FITC
0.511
0.621
P5-FITC
7.459
7.574
6.701
6.833
7.042
7.315
0.493
0.611
P4-FITC
9.247

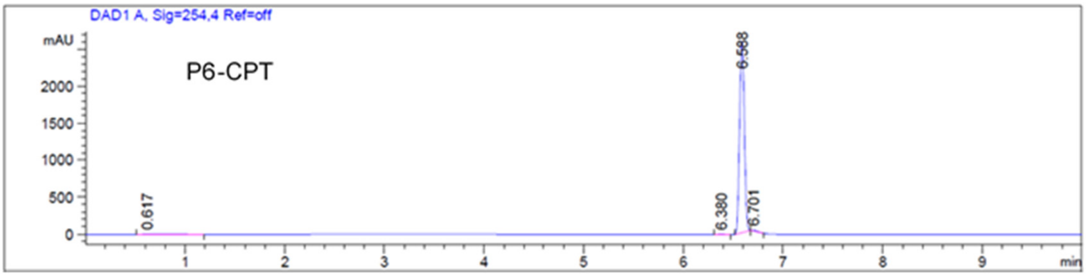
DAD1 A, Sig=254,4 Ref=off
P6-CPT
mAU
6.588
0.617
6.380
6.701
min

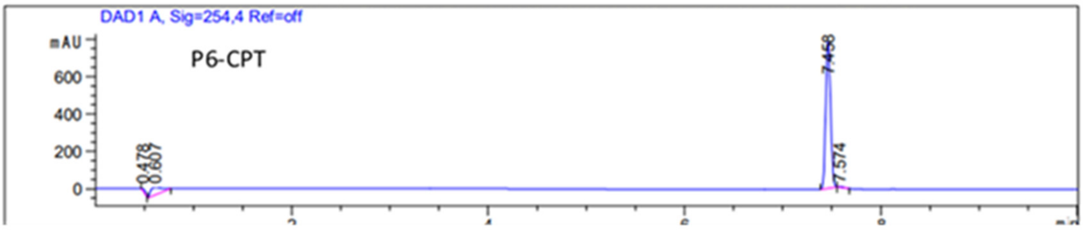
DAD1 A, Sig=254,4 Ref=off
P6-CPT
mAU
7.458
0.478
0.607
7.574

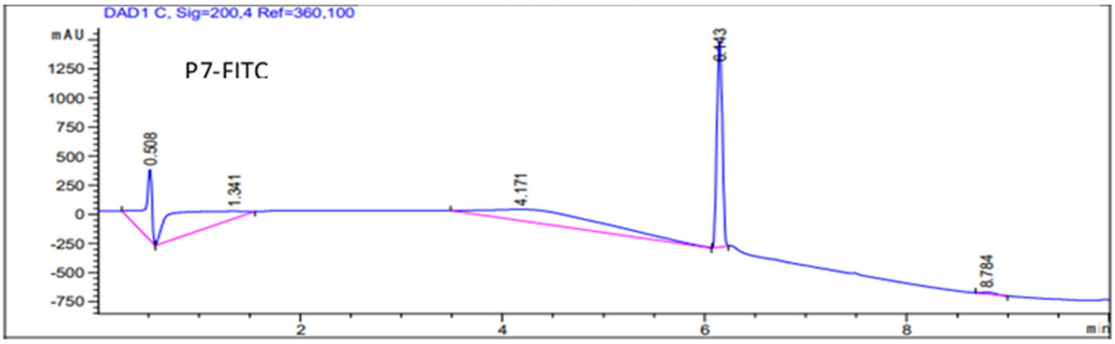
DAD1 C, Sig=200,4 Ref=360,100
P7-FITC
mAU
6.143
0.508
1.341
4.171
8.784
min

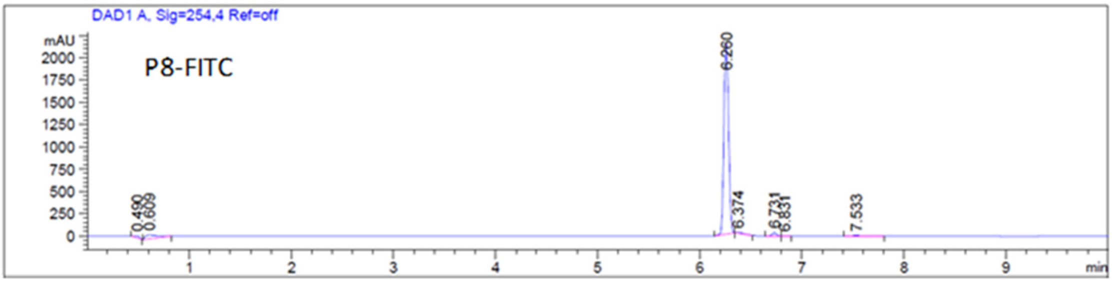
DAD1 A, Sig=254,4 Ref=off
P8-FITC
mAU
6.260
0.490
0.609
6.374
6.731
6.831
7.533
min

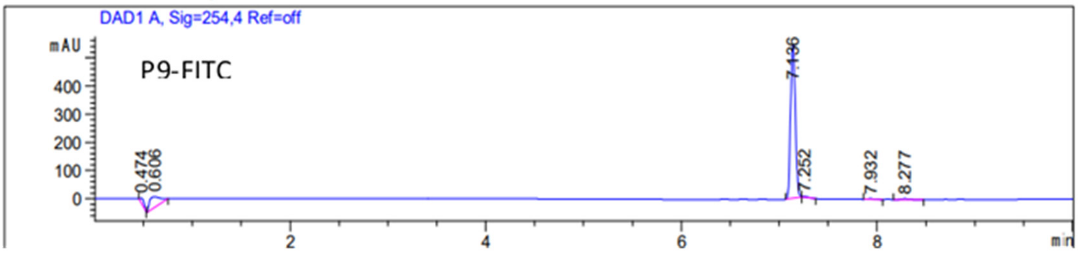
DAD1 A, Sig=254,4 Ref=off
P9-FITC
mAU
7.136
0.474
0.606
7.252
7.932
8.277
min

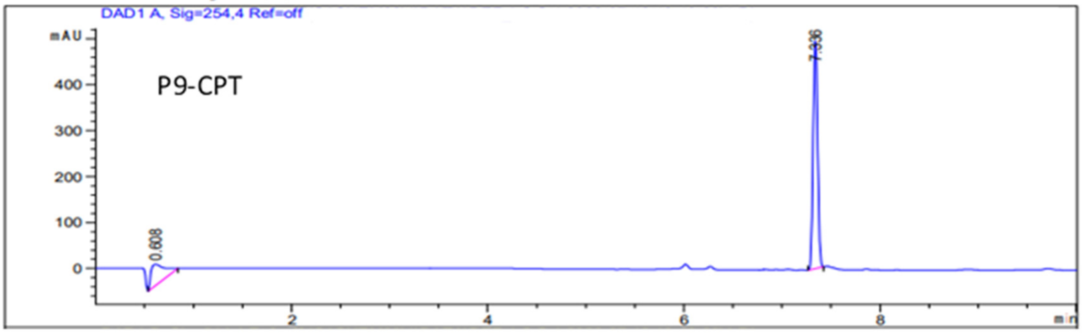
DAD1 A, Sig=254,4 Ref=off
P9-CPT
mAU
7.336
0.608
min

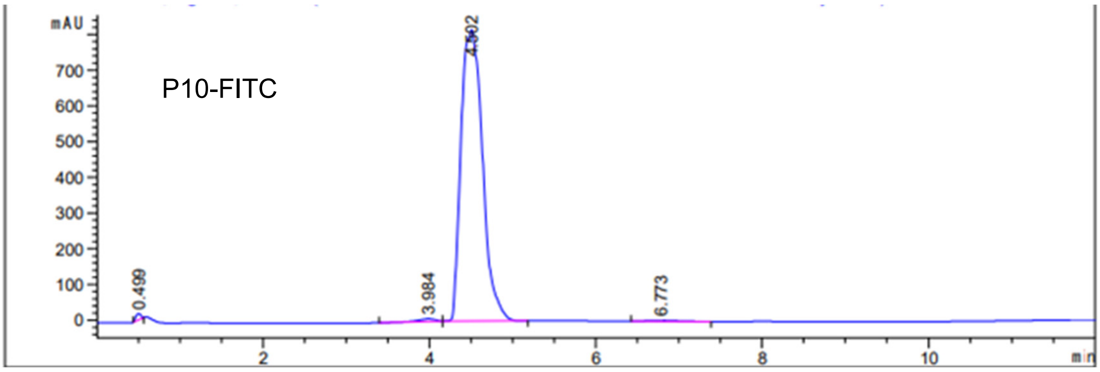
mAU
P10-FITC
4.502
0.499
3.984
6.773
min

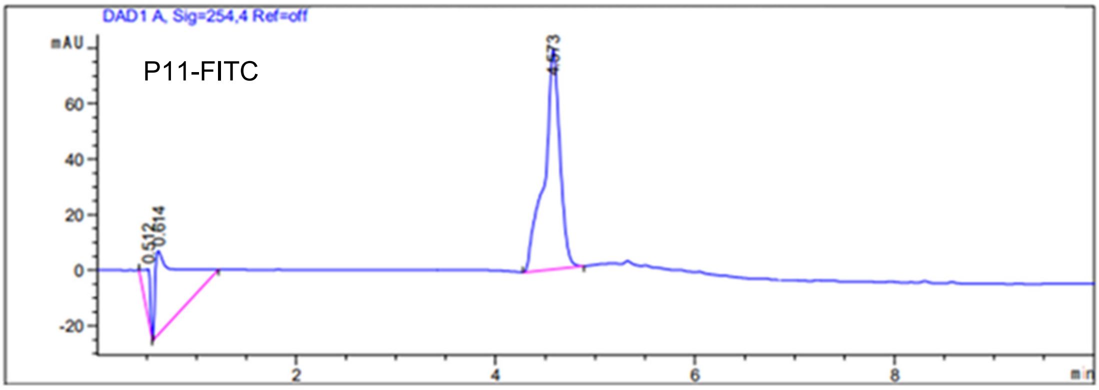
DAD1 A, Sig=254,4 Ref=off
mAU
P11-FITC
4.573
0.512
0.614
min
